# The impact of dieting culture is different between sexes in endurance athletes: a cross-sectional analysis

**DOI:** 10.1186/s13102-022-00549-4

**Published:** 2022-08-17

**Authors:** Austin J. Graybeal, Andreas Kreutzer, Jada L. Willis, Robyn Braun-Trocchio, Kamiah Moss, Meena Shah

**Affiliations:** 1 School of Kinesiology & Nutrition, College of Education and Human Sciences, University of Southern Mississippi, Hattiesburg, MS 39406 USA; 2 Department of Kinesiology, Harris College of Nursing & Health Sciences, Texas Christian University, Fort Worth, TX 76129 USA; 3 Department of Nutritional Sciences, College of Science & Engineering, Texas Christian University, Fort Worth, TX 76129 USA

**Keywords:** Sports nutrition, Female athlete nutrition, Endurance training, Disordered eating behaviors, Eating disorders

## Abstract

**Background:**

Frequent dieting is common in athletes attempting to achieve a body composition perceived to improve performance. Excessive dieting may indicate disordered eating (DE) behaviors and can result in clinical eating disorders. However, the current nutrition patterns that underly dieting culture are underexplored in endurance athletes. Therefore, the purpose of this study was to identify the sex differences in nutrition patterns among a group of endurance athletes.

**Methods:**

Two-hundred and thirty-one endurance athletes (females = 124) completed a questionnaire regarding their dieting patterns and associated variables.

**Results:**

The majority of athletes did not follow a planned diet (70.1%). For endurance athletes on planned diets (n = 69), males were more likely follow a balanced diet (*p* = 0.048) and females were more likely to follow a plant-based diet (*p* = 0.021). Female endurance athletes not on a planned diet (n = 162) were more likely to have attempted at least one diet (*p* < 0.001). Male athletes attempted 2.0 ± 1.3 different diets on average compared to 3.0 ± 2.0 for females (*p* = 0.002). Female athletes were more likely to attempt ≥ three diets (*p* = 0.022). The most common diet attempts included carbohydrate/energy restrictive, plant-based, and elimination diets. Females were more likely to attempt ketogenic (*p* = 0.047), low-carbohydrate (*p* = 0.002), and energy restricted diets (*p* = 0.010). Females made up the entirety of those who attempted gluten-/dairy-free diets (F = 22.0%, M = 0.0%).

**Conclusions:**

Being a female athlete is a major determinant of higher dieting frequency and continual implementation of popular restrictive dietary interventions. Sports dietitians and coaches should prospectively assess eating behavior and provide appropriate programming, education, and monitoring of female endurance athletes.

## Introduction

Body weight and composition are often viewed as critical performance variables in a variety of sports. [1] In some cases, athletes inherently possess or are genetically positioned to obtain a body composition specific to the demands of their sport; [2] however, many athletes participate in sports with body composition demands that are misaligned with their current anthropometry. [2] When athletes employ extreme dieting strategies to achieve a figure perceived to enhance performance, the misalignment between a sport-specific “ideal” and an athlete’s actual figure could promote disordered eating (DE) behaviors [3]. Further, athletes with a distorted perception of their current figure, also known as body dysmorphia, may also engage in DE behaviors despite possessing an ideal sport-specific body composition. [4] Under normal circumstances, dietary surveillance and food restriction may offer simplistic, focused, and successful dietary strategies. However, athletes, who also require anomalous dietary intakes to support their intensive training, are exposed to sport-specific factors associated with DE in addition to individual, social, cultural, biochemical, and genetic components. [2] As such, what may begin as a healthy attempt to achieve a body composition well-suited to the demands of a given sport often progresses toward chronic extreme dieting [2] and, over time, may manifest into clinical eating disorders. [3]

The term “dieting” is often used to reflect any shift in habitual dietary intake with a general focus on restricting food intake and excessive dietary surveillance. While the term has yet to be broadly defined, the socially influenced dichotomization (“on” or “off” a diet) and rigid surveillance of food that is reflected in eating behaviors is often referred to as “diet culture”. [5] Sport-specific dieting cultures are often cited in weight-class sports for reasons of regulation and tradition [1] as well as in aesthetic-sports if judges are influenced by a standard of thinness. [2] However, the thin ideal is also considered to be advantageous in endurance sports with common expressions such as “thin to win” or “runner’s body” that associate a specific somatotype with enhanced aerobic performance. [3, 6–8] This is, in part, due to the relationship between BMI and performance which supports that “lighter is better” for endurance events. [8] Thus, dieting cultures of endurance sports are often accepted by athletes, especially those with mismanaged programs or inappropriate coaching behavior. [9] Furthermore, endurance athletes employing rigid restriction and/or surveillance of food intake may be subjected to insufficient energy intake relative to exercise load, otherwise known as low energy availability (LEA). [10] Female athletes, specifically female runners and gymnasts, [11] are at a heightened risk for the consequences of LEA given that DE prevalence in female athletes are more than double that of male athletes; [3, 9, 12] although males with DE behaviors are also at risk for the consequences of LEA.

Sports nutrition is continually evolving as scientific research progresses and new fads are ever-present. The rapid shifts in sports nutrition trends combined with a surging diet culture in endurance sports may result in recurrent and potentially harmful dietary interventions. Because these trends change so frequently, the degree to which this occurs is unknown. While reports highlight dietary trends in endurance athletes, [13, 14] it is likely there are significant fluctuations and the continued investigation will further elucidate the dietary patterns of this group and improve the identification of potential DE behaviors. Therefore, the purpose of this study was to explore current and historical dieting patterns among a group of endurance athletes and if dieting frequency is more prominent in female endurance athletes using detailed questionnaires.

## Method

### Study design

A non-validated digital questionnaire was used to ask endurance athletes about their nutrition patterns. We recruited self-identified endurance athletes who were ≥ 18 years of age. Endurance athlete was defined as individuals who train and compete in long-duration aerobic exercise. Athletes in our sample performed endurance exercise training ≥ 3 d/wk with an average duration of 110.4 ± 54.6 min/training day. We recruited participants globally, via convenience sampling, using digital fliers and advertisements sent through email and various social media groups (i.e., Facebook groups, Twitter, Instagram) dedicated to endurance athletes and through word of mouth. Our study took place from June 2020 through February 2021 and was approved by the Texas Christian University Institutional Review Board (IRB # 1920-227). Informed consent was obtained before completing the survey. All responses were made anonymous. A total of 257 endurance athletes agreed to participate. Twenty-six were excluded for not providing critical demographic or other information.

### Questionnaire

Athletes completed a digital questionnaire using a personal device administered through Qualtrics (Survey Tool, Qualtrics® LLC, Provo, UT, USA). The questionnaire contained multiple choice, multiple select, and open-ended questions regarding participant demographics, sport history/participation, and dietary patterns. The initial question, “*Do you currently follow a diet*?” was used to determine overall prevalence. Display logic was used to produce a follow-up question where athletes who selected “*yes*” were provided a comprehensive list of planned diet types to select from which included both dietary archetypes and specific “named” or “branded” diets. In the instance that an athlete’s diet type was not listed, “*Other*” could be selected with fillable text for further description. If “*Other*” was selected, an investigator examined the fillable answer and allocated it to the appropriate section when necessary. If participants did not follow a planned diet, display logic produced the question “*Have you ever attempted to follow a diet*?”. Athletes who reported a previous diet attempt were provided the same comprehensive list of planned diet types including an option for “*Other*” and were asked to report all diets previously attempted. Athletes currently following a diet were asked to answer: 1) why they followed this diet, 2) what difficulties they had following this diet, and 3) how long they have been following this diet. Athletes who did not report following a diet were asked “*Why don’t you follow a planned diet?*” and those who reported a previous diet attempt were asked to answer: 1) why they attempted this diet(s), 2) why they discontinued this diet(s), 3) how long they were able to follow this diet(s) before discontinuing, and 4) if they were opened to trying a new diet strategy.

### Statistical analysis

Responses to the questions were reported as mean ± standard deviation or percent of the total. Participant characteristics by sex were analyzed using Chi-square (*Χ*^2^) and independent t-tests. Given the large number of possible responses to each question, responses with a prevalence of ≥ 20% were reported unless smaller distributions were necessary to explain larger themes. Responses by sex were analyzed using *Χ*^2^ and by independent t-tests. Yates correction for continuity were used for all 2 × 2 analyses. Nonparametric correlations were conducted to observe associations between participant characteristics and diet patterns using Spearman correlations. Logistic regression was used to predict group membership for diet patterns. For all logistic regression models, the categorial variables of sex, competition level, and primary sport and the continuous variable age were included. Hosmer–Lemeshow test showed goodness of fit for each model. Adjusted odds ratios (OR_a_) and corresponding 95% confidence intervals (95%CI) were calculated for all 2 × 2 analyses. Because large differences in sample size across primary sports skewed logistic regression analyses for some variables, unadjusted OR and corresponding 95%CI were conducted, when necessary, to examine group membership by sex only. Adjusted standard residuals (ASR) were used after initial *Χ*^2^ tests to determine differences from expected with a threshold of ± 2. Cohen’s d and corresponding 95%CI were reported when applicable. Kraemer’s V and phi (φ) were reported for *Χ*^2^. Statistical significance was set at *p* < 0.05. Data was analyzed using IBM SPSS version 26 (IBM, Armonk, NY).

## Results

### Participants

Participant characteristics are presented in Table 1. Males were significantly older (*p* = 0.011), taller (*p* < 0.001), weighed more (*p* < 0.001), had a higher BMI (*p* < 0.001), and trained more h/d (*p* = 0.016) than females. A greater number of cyclists were male and more runners were female (p_chi_ < 0.001, V = 0.38). More females were current collegiate athletes compared to males (p_chi_ = 0.045, V = 0.187). The sample was predominately Caucasian (88.7%) and non-Hispanic or Latino (87.8%).

**Table 1:** Characteristics of endurance athletes completing a nutrition questionnaire

	Total (%) n = 231	Male (%) n = 107	Female (%) n = 124
Male	107 (46.3%)		
Female	124 (53.7%)		
Cycling	47 (20.3%)	38 (35.3%)^a^	9 (7.3%)^b^
Running	82 (35.5%)	25 (23.4%)^b^	57 (46.0%)^a^
Triathlete	84 (36.4%)	37 (34.6%)	47 (37.9%)
Rowing	8 (3.5%)	3 (2.8%)	5 (4.0%)
Swimming	6 (2.6%)	2 (1.9%)	4 (3.2%)
Wheelchair	2 (0.9%)	1 (0.9%)	1 (0.8%)
Aqua bike	1 (0.4%)	1 (0.9%)	0 (0.0%)
Snowshoeing	1 (0.4%)	1 (0.9%)	0 (0.0%)
Age (y)	38.5 ± 13.3	40.8 ± 13.7^c^	36.4 ± 12.7
Height (cm)	171.4 ± 11.2	179.9 ± 7.9^c^	164.2 ± 8.1
Weight (kgs)	70.6 ± 16.3	81.8 ± 15.3^c^	61.1 ± 10.0
BMI (kg/m^2^)	23.9 ± 4.3	25.3 ± 4.7^c^	22.7 ± 3.7
Training h/d	1.8 ± 0.9	2.0 ± 1.0^c^	1.7 ± 0.9
Training d/wk	5.4 ± 1.3	5.2 ± 1.5	5.5 ± 1.2
Professional	11 (4.8%)	8 (7.5%)	3 (2.4)%
Collegiate	30 (13.0%)	8 (7.5%)^b^	22 (17.9%)^a^
Former Collegiate	36 (15.7%)	18 (16.8%)	18 (14.6%)
Recreational/ Amateur	153 (66.5%)	73 (68.2%)	80 (65.0%)
Top 3 Overall	49 (21.3%)	23 (21.7%)	26 (21.0%)
Top 3 Division	101 (43.7%)	40 (37.4%)	61 (49.2%)

Data presented as n (%) with differences determined by *X*^2^ or mean ± standard deviation with differences determined by independent t-tests and one-way ANOVA

^a^distribution greater than expected at *p* < 0.05

^b^distribution less than expected at *p* < 0.05

^c^significantly different than females *p* < 0.05

### Nutrition patterns and beliefs for athletes currently following a planned diet

Nutrition patterns for athletes currently following a planned diet are presented in Table 2. A total of 29.9% (n = 69) reported following a planned diet with no difference between groups (Males = 27.1%, Females = 32.3%, *p* = 0.48). Only balanced (cognizant of food choices and dietary intakes) and plant-based diets (e.g., vegetarian/vegan) had a prevalence of ≥ 20%. Male athletes were 4.4 times (95%CI: 1.0–19.3, *p* = 0.048) more likely to follow a balanced diet (p_chi_ = 0.28, φ = 0.16). Female athletes were 8.3 times (95%CI: 1.4–50.4, *p* = 0.021) more likely to follow plant-based diets (p_chi_ = 0.024, φ = 0.31) with an unadjusted OR of 5.2 (95%CI: 1.3–20.2, *p* = 0.017).

**Table 2:** Dietary practices of endurance athletes who reported following a planned diet

	Total (%) n = 69	Male (%) n = 29	Female (%) n = 40
What diet do you follow?			
Balanced diet	18 (26.1%)	10 (34.5%)	8 (20.0%)
Plant-based diet	18 (26.1%)	3 (10.3%)^b^	15 (37.5%)^a^
Why do you follow this diet?			
Health benefits	52 (75.4%)	22 (75.9%)	30 (75.0%)
Performance benefits	31 (44.9%)	19 (65.5%)^a^	12 (30.0%)^b^
Personal/medical reasons	31 (44.9%)	8 (27.6%)^b^	23 (57.5%)^a^
Do you have difficulty adhering to your diet?			
Yes/Occasionally	29 (42.0%)	12 (41.4%)	17 (42.5%)
No	40 (58.0%)	17 (58.6%)	23 (57.5%)
What difficulties do you experience?	n = 29	n = 12	n = 17
Social influences	12 (41.4%)	3 (25.0%)	9 (52.9%)
Preparing/obtaining food	9 (31.0%)	4 (33.3%)	5 (29.4%)
Scheduling conflicts	9 (31.0%)	5 (41.7%)	4 (23.5%)
General adherence	9 (31.0%)	4 (33.3%)	5 (29.4%)
How long have you been following this diet?^*^			
Less than a month	7 (10.3%)	3 (10.4%)	4 (10.3%)
Months	12 (17.6%)	9 (31.0%)	3 (7.7%)
Years	49 (72.1%)	17 (58.6%)^b^	32 (82.1%)^a^

Data presented as n (%) with differences determined by *X*^2^

^*^n(68)

^a^ distribution greater than expected at *p* < 0.05

^b^ distribution less than expected at *p* < 0.05

The most common reasons athletes reported following a diet were for health, performance, and personal/medical reasons. Male athletes were 9.8 times (95%CI: 2.4–40.0, *p* = 0.002) more likely to be following a diet for performance benefits (p_chi_ = 0.007, φ = 0.35) with an unadjusted OR of 4.4 (95%CI: 1.6–12.3, *p* = 0.004). Female athletes were 4.4 times (95%CI: 1.3–14.3, *p* = 0.022) more likely to follow a diet for personal/medical reasons (p_chi_ = 0.026, φ = 0.30). Most athletes employing a dietary strategy reported that they did not experience difficulties with adherence (58.0%). Of those that experienced difficulty either absolutely or occasionally, social influences (41.4%), preparing/obtaining foods (31.0%), scheduling conflicts (31.0%), and general adherence (31.0%) were reported as major obstacles. Lastly, when asked how long athletes had been following their diet, 72.1% reported *years*. Females were 7.2 times (95%CI: 1.3–41.16, *p* = 0.027) more likely to have been following their diet for years (p_chi_ = 0.027, φ = 0.33) with an unadjusted OR of 4.4 (95%CI: 1.3–15.7, *p* = 0.016).

### Nutrition patterns and beliefs for athletes not following a planned diet

Nutrition patterns for athletes not following a planned diet are presented in Table 3. Overall, 70.1% (n = 162) of athletes reported not following a specific diet strategy. Reasons for not following a specific diet included not wanting to follow one in general (39.5%) and that they were too difficult (25.3%). Of these athletes, 55.6% (n = 90) reported that they had previously attempted a diet strategy. Female athletes were 6.4 times (95%CI: 2.7–15.2, *p* < 0.001) more likely to have attempted at least one diet previously (p_chi_ < 0.001, φ = 0.31).

**Table 3:** Nutrition patterns of endurance athletes not following planned diet

	Total (%)	Male (%)	Female (%)
Have you ever attempted a diet?	n = 162	n = 78	n = 84
Yes	90 (55.6%)	31 (39.7%)^b^	59 (69.2%)^a^
How many diets have you attempted? (n = 90)	n = 90	n = 31	n = 59
Different diets attempted	2.8 ± 1.9	2.0 ± 1.3	3.0 ± 2.0^c^
Attempted ≥ 3 diets	38 (42.2%)	6 (19.4%)^b^	32 (54.2%)^a^
What diet(s) did you attempt?			
Ketogenic/Atkins/Low-carbohydrate	46 (51.1%)	13 (41.9%)	33 (55.9%)
Balanced diet	34 (37.8%)	12 (38.7%)	22 (37.3%)
Low-carbohydrate only	29 (32.2%)	5 (16.1%)^b^	24 (40.7%)^a^
Energy restricted	27 (30.0%)	3 (9.7%)^b^	24 (40.7%)^a^
Paleo/Whole-30	27 (30.0%)	9 (29.0%)	18 (30.5%)
Plant-based diet	25 (27.8%)	6 (19.4%)	19 (32.2%)
Ketogenic diet only	23 (25.6%)	6 (19.4%)	17 (28.8%)
Intermittent fasting	19 (21.1%)	4 (16.1%)	15 (23.7%)
Gluten/Diary-free	13 (14.4%)	0 (0.0%)	13 (22.0%)
Why did you stop following that diet?			
Difficulty following	35 (38.9%)	13 (37.3%)	22 (37.3%)
Can’t eat foods I enjoy	25 (27.8%)	6 (19.4%)	19 (32.2%)
Difficult to obtain/prepare food	23 (25.6%)	6 (19.4%)	17 (28.8%)
Personal/Medical reason	19 (21.1%)	2 (6.5%)^b^	17 (28.8%)^a^
How long were you able to follow the diet before stopping?			
Less than 1 wk	15 (16.7%)	5 (16.1%)	10 (16.9%)
1 wk–less than 1 mo	37 (41.1%)	17 (54.8%)	20 (33.9%)
1 mo–less than 3 mo	18 (20.0%)	3 (9.7%)	15 (25.4%)
3 mo–less than 1 yr	17 (18.9%)	6 (19.4%)	11 (18.6%)
Greater than 1 yr	3 (3.3%)	0 (0.0%)	5 (5.1%)

Data presented as mean ± standard deviation with differences determined by independent t-tests or n (%) with differences determined by *X*^2^

^a^ distribution greater than expected at *p* < 0.05

^b^ distribution lower than expected at *p* < 0.05

^c^ significantly different at *p* < 0.05

Among those who had previously attempted at least one diet, balanced, energy restricted, low-carbohydrate, ketogenic, intermittent fasting, plant-based, and paleo/Whole30 were the most frequently reported. Female athletes were 5.6 times (95%CI: 1.0–30.4, *p* = 0.047) more likely to follow a ketogenic diet (p_chi_ = 0.47, φ = 0.103). Female athletes were also 43.0 times (95%CI: 3.9–469.8, *p* = 0.002) more likely to follow a low-carbohydrate diet. Given the high degree of skewness in the OR_a_ for low-carbohydrate diets, an unadjusted OR was conducted and revealed that female athletes were 3.6 times (95%CI: 1.2–10.6, *p* = 0.022) more likely to follow a low-carbohydrate diet which was further supported by X^2^ (p_chi_ = 0.033, φ = 0.25). When low-carbohydrate diet strategies were combined (ketogenic, Atkins, low-carbohydrate) female athletes were 10.3 times (95%CI: 1.8–57.1, *p* = 0.008) more likely to follow this diet type with an unadjusted OR of 1.8 (95%CI: 0.7–4.2, *p* = 0.21). Female athletes were 18.5 times (95%CI: 2.0–172.4, *p* = 0.010) more likely to follow energy restricted diets. An unadjusted OR revealed that females were 6.4 times (95%CI: 1.8–23.5, *p* = 0.005) more likely to follow energy-restricted diets which was further supported by X^2^ (p_chi_ = 0.005, φ = 0.32). Females made up the entirety of those who previously followed a dairy- and gluten-free diet (22.0%) which did not allow for a comparison with male athletes (0.0%). Athletes reported abandoning their diet strategy due to general difficulty, inability to eat enjoyable foods, difficulty obtaining/preparing foods, and personal/medical reasons. It was more common for females to abandon their diet for personal/medical reasons (p_chi_ = 0.028, φ = 0.26). When asked how long they were able to follow their diet(s) before stopping, 57.8% of athletes reported a duration of less than one month and 77.8% less than three months. However, 90.0% reported that they would consider attempting another diet.

The average number of summed diet attempts was 2.76 (95%CI: 2.36–3.15) with a minimum of 1 and maximum of 10 (Male: min = 1, max = 6; Female: min = 1, max = 10). On average, males (2.0 ± 1.3) had significantly less diet attempts compared to females (Females = 3.0 ± 2.0, *p* = 0.002; d = − 0.64, d^95%CI^: − 1.1, − 0.2). Nonparametric correlations were significant for total diet attempts and sex (Spearman r = 0.31, *p* = 0.003). Interestingly, of those that reported attempting a diet previously, 42.2% reported attempting ≥ three diets and females athletes were 4.5 times (95%CI: 1.2–16.2, *p* = 0.022) more likely to have attempted this many diets (p_chi_ = 0.003, φ = 0.34).

## Discussion

Our study sought to identify sex-related differences in dieting patterns in a group of endurance athletes. This is the first study, to our knowledge, to fully encompass the persistent sex-related differences in planned dietary patterns, providing evidence toward the influence of diet culture in female endurance athletes. The major findings of our study were: 1) female endurance athletes were more likely to have attempted and discontinued a planned diet strategy previously and have a greater number of total diet attempts; 2) female endurance athletes were more likely to follow carbohydrate and energy restricted diets, plant-based diets, and elimination diets (ex: gluten/dairy-free); and 3) female endurance athletes were more likely to follow, currently or previously, a planned diet for personal reasons compared to males who’s motivation appeared more performance oriented.

Manipulation of one’s body mass is a common component of many endurance sporting events. For instance, competitive cyclists may attempt to lose weight leading up to competition to improve power-to-weight ratio. [15] Marathon runners may also desire a lower body mass to increase running speed during competition. [16] Although body weight alterations are common in endurance sports, less is known about the dieting strategies endurance athletes employ to achieve an ideal body composition, weight, or both. While these strategies appear advantageous and often result in initial success, continual efforts to replicate a specific body composition or performance outcome may lead to DE behaviors. [3, 7] However, determining whether an endurance athlete has an increased risk for DE is difficult due to the dieting culture integrated into endurance sports, specifically for female athletes. Professional organizations suggest that unhealthy and persistent dietary behaviors represent an increased risk for eating disorders. [17] Thus, quantifying dieting frequency may be an initial indicator of DE behaviors in endurance athletes.

Notably, our study objectively measured dieting frequency and history, showing that ~ 30% of endurance athletes were currently following a planned diet and of those not currently following a planned diet, 55.6% had attempted to follow at least one diet previously. While there are few studies [13, 14] that report dieting frequency or history in a sample of heterogenous endurance athletes, the combined frequency observed in our study is similar to that reported in elite Olympic weight-class athletes. [18] Specifically, our study showed that female endurance athletes were more likely to attempt and discontinue a planned dietary strategy, more likely to have attempted ≥ three different dietary strategies, and had a significantly greater number of planned diet attempts compared to male endurance athletes. The extensive dieting frequency in female endurance athletes supports the persistent component of DE evidenced by studies in athletes showing associations between dieting and eating behaviors. [19] Thus, collecting diet frequency prior to programming may assist coaches and sports dietitians in early detection of DE for female endurance athletes.

Shifts in sports nutrition have recently focused on personalized dietary prescription. [20] However, appropriately individualized dietary prescriptions are often only available to those with access to nutrition professionals. Those without access to nutrition professionals, such as the recreational endurance athletes in our study, are more reliant on coaches and media for diet prescription. [21] As a result, popular diets that receive considerable media attention are commonly employed. This may be problematic given that endurance athletes require greater energy intake, but most popular diets promote severe restriction of at least one macronutrient, total energy, and/or entire food items or categories. Though, the extent to which endurance athletes follow these diets has been underexplored.

The findings from our study show that female endurance athletes are more likely to follow carbohydrate restriction diets. A common reason for carbohydrate restriction in athletes is to increase the body’s ability to use fat as the primary energy source at exercise intensities where it would normally use glycogen. The transition from carbohydrate to fat utilization at higher exercise intensities promotes glycogen sparing and improved makers of aerobic performance; however, this has not shown to improve competition outcomes. [22] Although there is theoretical rationale for employing carbohydrate restriction in endurance sports, the prevalence of previous carbohydrate restriction in our group of female athletes is likely because female athletes in our study were predominately runners. As previously discussed, thinner figure is often more desirable for runners. [8] Female runners may also be more concerned that a larger proportion of carbohydrates in their diet could lead to increases in fat mass. The degree of carbohydrate intake proposed for endurance athletes may also cause hesitation due to anticipation of future weight gain. Irrespective of sport, females tend to report a greater concern for carbohydrate intake compared to males [23] which was supported by our adjusted analyses. Regardless, severe carbohydrate restriction may be attractive to female endurance athletes given the diet’s ability to produce rapid weight loss. However, there are many concerns with persistent carbohydrate restriction. Several studies report the appetite suppressive effects of carbohydrate restriction diets. [24] Because endurance athletes require sufficient energy to support their training, diets that reduce appetite in conjunction with decreased energy intake from acute exercise-induced anorexia may lead to LEA [25]. Other concerns with low-carbohydrate diets include the rapid reduction in markers of bone health, [26] hypercholesterolemia, [27] and nutrient deficiencies. [20]

Carbohydrate restriction often coincides with general energy restriction given that the near-complete removal of carbohydrates is difficult to replace. This is supported by our finding that female athletes showed more persistent attempts at both carbohydrate and energy restriction diets. Energy restriction diets were higher in female athletes and female athletes had a lower BMI than male athletes. The emphasis on energy restriction despite lower BMI may indicate DE behaviors that are coupled with (or originate from) a hyper fixation on achieving a sport-specific body type. [6] Given the high energy requirements necessary to support endurance exercise and that female athletes habitually consume less energy than they expend, [28] it is difficult to recommend continual energy restriction to female endurance athletes. Sufficient energy intake is critical in bone mineral formation, reproductive health [29], and limiting common nutrient deficiencies observed in female athletes. [24] Moreover, Coelho et al. [7] suggests that athletes at the highest risk for eating disorders are those who restrict energy intake.

Coelho et al. [7] also suggests that vegetarian athletes are amongst those with a higher risk for the development of eating disorders. In our study, it was more common for female athletes to follow plant-based diets compared to male athletes. This trend was observed in females currently on a planned diet and those with previous diet attempts and was similar to what has been reported in elite athletes [30]. Interestingly, female athletes were more likely to follow a plant-based diet for personal reasons. Given that most athletes currently following a planned diet reported continuous implementation of their strategy, it is possible that social/cultural (animal-activism, agricultural concerns, cultural and/or religious restriction, etc.) influences made up a considerable number of these personal reasons. Orthorexia nervosa, characterized as an obsession with healthy eating that is associated with restrictive behaviors, [31] may be another reason female athletes are more likely to follow a plant-based diet. In other words, because dietary restrictions are common in orthorexia nervosa as a result of this obsession, there could be a crossover between orthorexia nervosa (restricting dietary intakes to only “healthy” foods) and plant-based diets (restricting dietary intakes to only “plant-based” food which are often considered to be “healthy”). While orthorexia nervosa may not be initially associated with sport-specific DE, excessive exercise combined with an obsession to restrict “unhealthy” foods certainly corresponds with DE behaviors. Interestingly, male athletes were more likely to follow a balanced diet suggesting that they did not share a similar obsession with the removal of specific foods or rigid surveillance. On the other hand, in those not currently following a planned diet, the attempt and rapid discontinuation of a plant-based diet refutes the aforementioned motivations (personal reasons, orthorexia nervosa, etc.) and thus, are likely more aligned with achieving an ideal figure. Similar to low-carbohydrate strategies, plant-based diets have several benefits for endurance athletes. For instance, plant-based diets have shown to increase muscle glycogen stores, delay fatigue, and reduce inflammation and oxidative stress when energy intake is sufficient. [20] However, similar to other restrictive diets, athletes following vegetarian diets are at a higher risk for micronutrient decencies and LEA. [32]

Although we were unable to conduct an inferential analysis, female endurance athletes were more likely to attempt either a diary- or gluten-free diet. Endurance runners frequently experience exercised-induced gastrointestinal symptoms and often avoid particular foods. [33] The elimination of dairy and gluten from diet may be a common approach for endurance athletes to alleviate gastrointestinal issues with or without a known intolerance. However, reports show no sex-related differences in the frequency of gastrointestinal symptoms following endurance exercise. [34] If the reasons for employing dairy- and gluten-free diets were solely associated with gastrointestinal issues, the proportion would theoretically be more balanced between sexes. Thus, it is plausible to consider that the motivation to follow dairy- and/or gluten-free diets likely aligns more with orthorexia nervosa than gastrointestinal health for female athletes. In fact, the primary risks of employing a dairy- or gluten-free diet align with LEA and micronutrient and fiber deficiencies [32]. However, it is unknown if these diets alone result in chronic LEA.

Our data suggest that female endurance athletes are subjected to the diet culture of endurance sports to a greater degree than their male counterparts by way of increased dieting frequency and diet selection and may be at a greater risk for developing eating disorders associated with endurance sports thus supporting our hypothesis. Despite the risks associated with many popular diets, it is certainly possible to employ them effectively if closely monitored. However, most attempted diets appeared to create considerable burden and the majority were abandoned within three months. While it is unclear exactly what contributes to such unsuccessful diet attempts in endurance athletes, the restrictive nature of popular diets may be too difficult for long-term implementation. However, despite the reported obstacles, we still observed a high dieting frequency and an openness to future diet attempts. Thus, the implementation of frequent and restrictive diets is likely to remain and coaches and sports dietitians should prioritize screening and surveillance during programming and prioritizing fueling for training.

The current study had several limitations that warrant discussion. First, our study was based on self-report which may lead to inaccuracies in reporting, especially considering that the questionnaire was non-validated. Additionally, the cross-sectional nature of the study may have resulted in dietary practices that differ from general intakes given that practices vary by training/competition cycles. However, our study was conducted across a nine-month period which may have accounted for differences by season although this was not included in the analysis. Future studies should determine the dieting patterns of endurance athletes across timepoints within an athletic season. The study period also took place during the COVID-19 pandemic and responses may not have been indicative of habitual intake. However, it is unlikely that COVID-19 significantly influenced total dietary history. Our sample also had uneven proportions of professional, collegiate, and amateur endurance athletes. However, our sample was mostly amateur athletes, followed by collegiate, and professional and likely similar to the true prevalence of endurance athletes. Our study also did not ask for subcategories of many branded diets (i.e., adapted ketogenic diet, ovo-vegetarian, etc.) which may have revealed other dietary patterns. However, athletes were instructed to report and describe diet combinations, diet sub-categories, and diets that were not listed as “other”. Our study did not ask about dieting history in those currently following a planned diet. However, our results indicate that the majority of these endurance athletes sustained their practice for at least one year.

## Conclusions

In conclusion, being a female endurance athlete is a major determinant of higher dieting frequency and recurrent implementation of popular restrictive dietary interventions; both of which may indicate an increased risk for current or future eating disorders. Collectively, our data show that dieting culture in endurance sports is apparent in female endurance athletes and may result in greater susceptibility for harmful psychological and physiological effects of DE. Therefore, sports dietitians and coaches should prospectively assess eating behavior and provide appropriate programming, education, and monitoring of female endurance athletes. Prospective identification may allow coaches and sports dietitians to better manage programs and recommend counseling or other support services before DE behaviors manifest into clinical eating disorders. Future research should investigate the effects of well-planned popular dietary interventions to determine safe and effective methods of employing common strategies and conduct further research on male athletes.

## Data Availability

Data is available upon reasonable request. Please contact Austin J. Graybeal, PhD, CSCS, Assistant Professor, School of Kinesiology & Nutrition, College of Education and Human Sciences, University of Southern Mississippi, Hattiesburg, MS 39406, USA. Phone: 601-266-5996. Email address: austin.graybeal@usm.edu.

## Abbreviations

BMI: Body mass index
DE: Disordered eating
LEA: Low energy availability
OR: Odds ratio

## References

[1] Ackland TR, Lohman TG, Sundgot-Borgen J, Maughan RJ, Meyer NL, Stewart AD (2012). Current status of body composition assessment in sport. Sports Med.

[2] Sundgot-Borgen J, Meyer NL, Lohman TG, Ackland TR, Maughan RJ, Stewart AD (2013). How to minimise the health risks to athletes who compete in weight-sensitive sports review and position statement on behalf of the Ad Hoc research working group on body composition, health and performance, under the auspices of the IOC Medical commission. Br J Sports Med.

[3] Sundgot-Borgen J, Torstveit MK (2010). Aspects of disordered eating continuum in elite high-intensity sports. Scand J Med Sci Sports.

[4] Kristjánsdóttir H, Sigurðardóttir P, Jónsdóttir S, Þorsteinsdóttir G, Saavedra J (2019). Body image concern and eating disorder symptoms among elite Icelandic athletes. Int J Environ Res Public Health.

[5] Jovanovski N, Jaeger T (2022). Demystifying ‘diet culture’: exploring the meaning of diet culture in online ‘anti-diet’ feminist, fat activist, and health professional communities. Womens Stud Int Forum.

[6] Anderson LM, Reilly EE, Gorrell S, Anderson DA (2016). Running to win or to be thin? An evaluation of body dissatisfaction and eating disorder symptoms among adult runners. Body Image.

[7] de Coelho GM, da Gomes AIS, Ribeiro BG, de Soares AE (2014). Prevention of eating disorders in female athletes. Open Access J Sports Med.

[8] Sedeaud A, Marc A, Marck A, Dor F, Schipman J, Dorsey M (2014). BMI, a performance parameter for speed improvement. PLoS One.

[9] Mountjoy M, Sundgot-Borgen J, Burke L, Carter S, Constantini N, Lebrun C (2014). The IOC consensus statement: beyond the female athlete triad–relative energy deficiency in sport (RED-S). Br J Sports Med.

[10] Loucks AB, Maughan Ronald J (2013). Energy balance and energy availability. The encyclopaedia of sports medicine.

[11] Schaal K, Tafflet M, Nassif H, Thibault V, Pichard C, Alcotte M (2011). Psychological balance in high level athletes: gender-based differences and sport-specific patterns. PLoS One.

[12] Martinsen M, Sundgot-Borgen J (2013). Higher prevalence of eating disorders among adolescent elite athletes than controls. Med Sci Sports Exerc.

[13] Baranauskas M, Stukas R, Tubelis L, Žagminas K, Šurkienė G, Švedas E (2015). Nutritional habits among high-performance endurance athletes. Med Kaunas Lith.

[14] Papadopoulou SK, Xyla EE, Methenitis S, Feidantsis KG, Kotsis Y, Pagkalos IG (2018). Nutrition strategies before and during ultra-endurance event: a significant gap between science and practice. Scand J Med Sci Sports.

[15] Ferguson LM, Rossi KA, Ward E, Jadwin E, Miller TA, Miller WC (2009). Effects of caloric restriction and overnight fasting on cycling endurance performance. J Strength Cond Res.

[16] Nikolaidis PT, Rosemann T, Knechtle B (2021). Development and validation of prediction equation of “Athens authentic marathon” men’s race speed. Front Physiol.

[17] Ljungqvist A, Jenoure P, Engebretsen L, Alonso JM, Bahr R, Clough A (2009). The international olympic committee (IOC) consensus statement on periodic health evaluation of elite athletes March 2009. Br J Sports Med.

[18] Sundgot-Borgen J, Garthe I (2011). Elite athletes in aesthetic and olympic weight-class sports and the challenge of body weight and body compositions. J Sports Sci.

[19] Martinsen M, Bratland-Sanda S, Eriksson AK, Sundgot-Borgen J (2010). Dieting to win or to be thin? a study of dieting and disordered eating among adolescent elite athletes and non-athlete controls. Br J Sports Med.

[20] Devrim-Lanpir A, Hill L, Knechtle B (2021). Efficacy of popular diets applied by endurance athletes on sports performance: beneficial or detrimental? a narrative review. Nutrients.

[21] Braun-Trocchio R, Graybeal AJ, Kreutzer A, Warfield E, Renteria J, Harrison K (2022). Recovery strategies in endurance athletes. J Funct Morphol Kinesiol.

[22] Burke LM, Ross ML, Garvican-Lewis LA, Welvaert M, Heikura IA, Forbes SG (2017). Low carbohydrate, high fat diet impairs exercise economy and negates the performance benefit from intensified training in elite race walkers. J Physiol.

[23] Davy SR, Benes BA, Driskell JA (2006). Sex differences in dieting trends, eating habits, and nutrition beliefs of a group of midwestern college students. J Am Diet Assoc.

[24] Gibson AA, Seimon RV, Lee CMY, Ayre J, Franklin J, Markovic TP (2015). Do ketogenic diets really suppress appetite? A systematic review and meta-analysis. Obes Rev.

[25] Graybeal AJ, Kreutzer A, Rack P, Moss K, Augsburger G, Willis JL (2021). Perceptions of appetite do not match hormonal measures of appetite in trained competitive cyclists and triathletes following a ketogenic diet compared to a high-carbohydrate or habitual diet: a randomized crossover trial. Nutr Res.

[26] Heikura IA, Burke LM, Hawley JA, Ross ML, Garvican-Lewis L, Sharma AP (2020). A short-term ketogenic diet impairs markers of bone health in response to exercise. Front Endocrinol.

[27] Creighton BC, Hyde PN, Maresh CM, Kraemer WJ, Phinney SD, Volek JS (2018). Paradox of hypercholesterolaemia in highly trained, keto-adapted athletes. BMJ Open Sport — Exerc Med.

[28] Beals KA (2002). Eating behaviors, nutritional status, and menstrual function in elite female adolescent volleyball players. J Am Diet Assoc.

[29] Nattiv A, Loucks AB, Manore MM (2007). The female athlete triad. Med Sci Sports Exerc.

[30] Heikura IA, Stellingwerff T, Burke LM (2018). Self-reported periodization of nutrition in elite female and male runners and race walkers. Front Physiol.

[31] Scarff JR (2017). Orthorexia nervosa: an obsession with healthy eating. Fed Pract.

[32] Cialdella-Kam L, Kulpins D, Manore MM (2016). Vegetarian, gluten-free, and energy restricted diets in female athletes. Sports.

[33] Parnell JA, Wagner-Jones K, Madden RF, Erdman KA (2020). Dietary restrictions in endurance runners to mitigate exercise-induced gastrointestinal symptoms. J Int Soc Sports Nutr.

[34] Snipe RMJ, Costa RJS (2018). Does biological sex impact intestinal epithelial injury, small intestine permeability, gastrointestinal symptoms and systemic cytokine profile in response to exertional-heat stress?. J Sports Sci.

